# Proteins interacting with *Leishmania major* PUF1: A proteomic dataset

**DOI:** 10.1016/j.dib.2020.106594

**Published:** 2020-11-26

**Authors:** África Sanchiz, Darío López-García, Carlos García-García, Irene Ozaez, Begoña Aguado, Jose María Requena

**Affiliations:** Centro de Biología Molecular “Severo Ochoa” (CBMSO, CSIC-UAM) Campus de Excelencia Internacional (CEI) UAM+CSIC, Universidad Autónoma de Madrid, 28049 Madrid, Spain

**Keywords:** Interactome, Co-immunoprecipitation, PUF1 antibody, *Leishmania major*, Proteomics

## Abstract

*Leishmania* parasites must deal with stressful environmental conditions (thermal, nutritional and oxidative) along their digenetic life cycles. This requires drastic changes in gene expression, which in this parasite occurs mainly through post-transcriptional mechanisms involving RNA binding proteins (RBPs). PUF proteins, a class of RBPs existing in most eukaryotic organisms, might play too an essential role modulating the fate of mRNAs and regulating gene expression in *Leishmania* parasites. A proteomic approach to identify putative protein partners (interactome) of the *Leishmania major* PUF1 protein was performed. The PUF1 interactome was characterized by co-immunoprecipitation using *L. major* cellular extracts and an anti-LiPUF1 antibody, and a subsequent analysis of the co-immunoprecipitated proteins by mass-spectrometry, identifying 90 LmPUF1 candidate partners. Remarkably, many of the identified proteins are other RBPs and/or putative P bodies and mRNA-exporting machinery components. Raw mass spectrometry data are available via ProteomeXchange with identifier PXD022581.

## Specifications Table

**Table:** 

Subject	Biochemistry, Genetics and Molecular Biology (General)
Specific subject area	*Leishmania major* proteomics
Type of data	Figures Supplementary tables in Excel file Proteome raw data
How data were acquired	Instruments: Liquid Chromatography-MS/MS, LTQ-Orbitrap Velos. Software: PEAKS X+ and *L. major* protein database from Uniprot (date of access: 30/09/19).
Data format	Raw, filtered and analysed
Parameters for data collection	Reverse phase- Liquid Chomatography (RP-LC)/Mass spectrometry (MS) analysis of proteins from *L. major* extracts immunoprecipitated by an anti-PUF1 antibody
Description of data collection	Protein extracts from *L. major* promastigotes were incubated with an affinity purified anti-LiPUF1 antibody. Afterwards, immunocomplexes were selected using Dynabeads Protein A beads (Invitrogen). Samples (three replicates and two controls) were submitted to trypsin digest and LC-MS/MS analysis for protein identification.
Data source location	Proteomics Facility Institution: Centro de Biologia Molecular Severo Ochoa (CSIC-UAM), Universidad Autonoma de Madrid City: Madrid Country: Spain
Data accessibility	Raw MS files were deposited in ProteomeXchange (PXD022581) [DOI: 10.6019/PXD022581]. Processed datasets are within the article, and additional details are provided in the supplementary Excel file

## Value of the Data

• Protein immunoprecipitation using a specific antibody against *Leishmania* PUF1 and the analysis of the immunocomplexes via LC-MS/MS allowed the identification of potential PUF1 interactors in *L. major* promastigotes.
• The identification of proteins interacting with PUF proteins, in this case with PUF1, provides clues about the *Leishmania* cellular processes in which these proteins are involved. Also, these data might be useful for understanding the role played by PUF1 in related parasites like *Trypanosoma brucei* and *Trypanosoma cruzi*, which possess a similar set of PUF proteins and share gene expression regulatory mechanisms.
• These data can be used for researchers to look whether particular proteins were present in this dataset. In this regards, it should be noted that some of the proteins identified in this study may be interacting indirectly with PUF1, as no RNAse treatment of the samples was performed, and, therefore, some proteins may be present in the dataset by its binding to the PUF1-bound RNAs.
• Apart from the identification of 90 candidate proteins for their statistically significant association with PUF1, this dataset contains peptide data for 626 proteins of *L. major*. In *Leishmania*, due to their evolutionarily divergence from model eukaryotes, many of their putative proteins have been predicted from the genome sequence and annotated as hypothetical proteins, because of the lack of clear orthology with known proteins. In this dataset, researchers can find a demonstration of a real existence for some of those hypothetical proteins.

## 1 Data Description

**Fig. 1:**
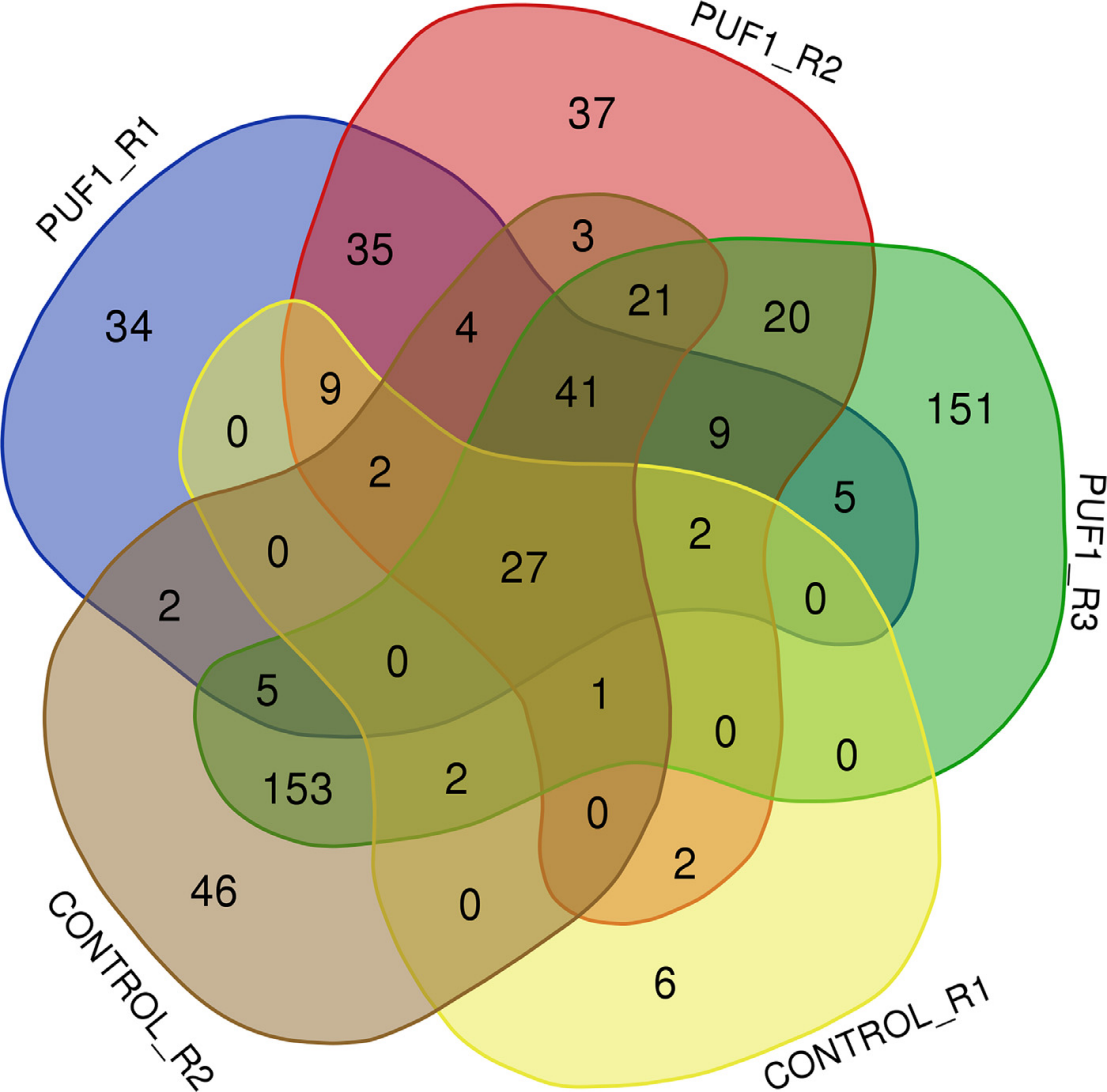
Venn diagram showing the numbers of coincident proteins identified in each of the five assays. PUF1_R1, _R2, and _R3 corresponds to proteins identified in the three immunoprecipitations using the anti-LiPUF1 antibody, whereas controls (CONTROL_R1 and _R2) corresponds to the proteins identified using the pre-immune serum. For protein identification, False Discovery Rate was set to 0.01 and only proteins identified by two or more unique peptides were considered.

Five independent immunoprecipitation assays were performed: three using a specific anti-PUF1 antibody and two controls, using the pre-immune serum. The immunoprecipitated proteins were analysed by Reverse phase-liquid chromatography RP-LC-MS/MS analysis. The proteins identified in each individual experiment are listed in Supplementary Table S1 (Excel file with different sheets for each assay). A graphical representation of coincident and non-coincident proteins among the five data sets is shown in Fig. 1.

Additionally, supplementary Table S2 lists the peptides that allowed the identification of every protein, also separated in different sheets according to each individual assay. Finally, supplementary Table S3 lists the 90 proteins found, after statistical analysis, to be specifically co-immunoprecipitated together with *L. major* PUF1 (LmjPUF1); these proteins would constitute part of the LmjPUF1 interactome. For determining the specificity, a binomial statistical test was used considering the sum of peptides identified for every protein in the anti-PUF1 assays versus the control assays. Only proteins with *p* values lower than 0.05 were considered as putative components of the PUF1 interactome.

**Fig. 2:**
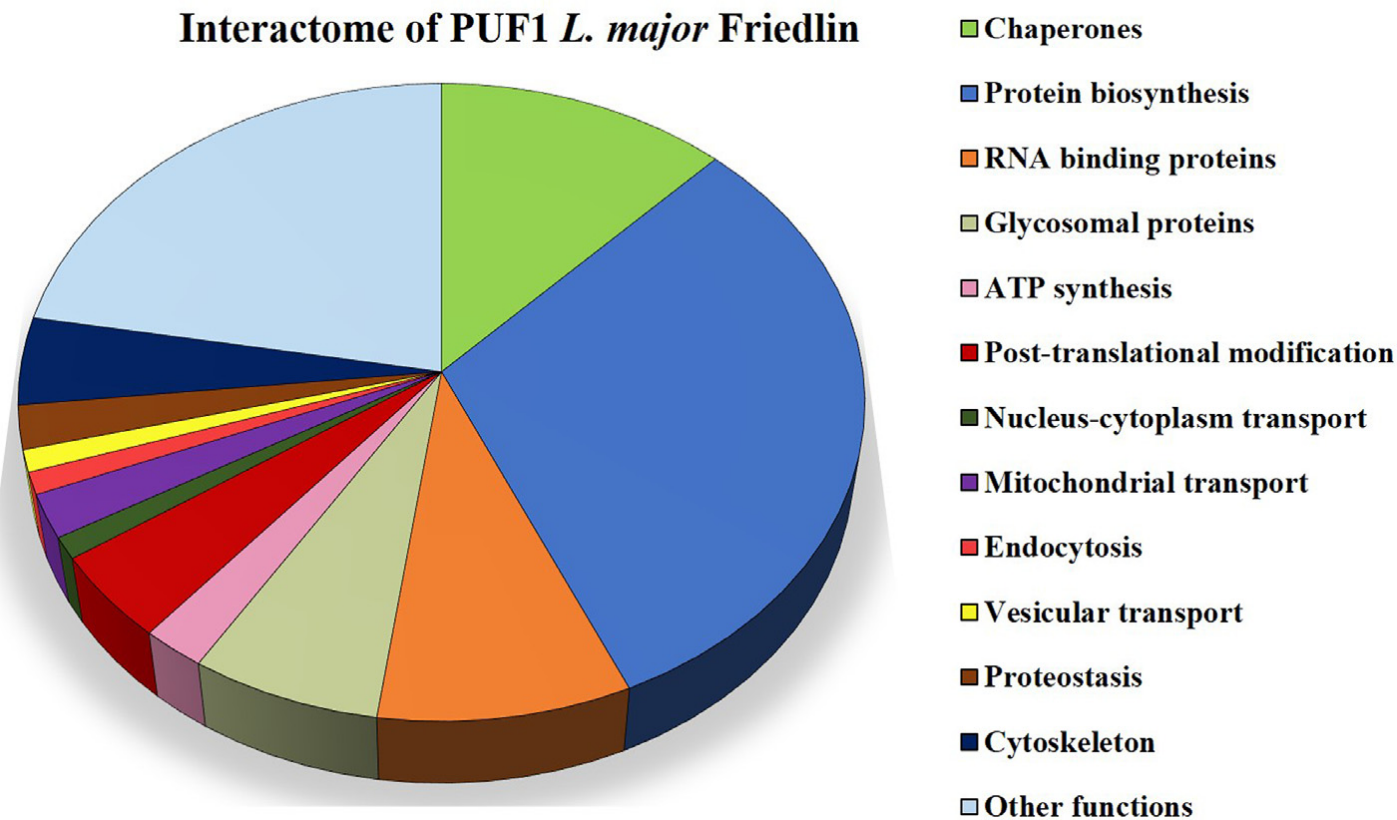
Functional classification of the 90 protein identified as candidate partners for LmjPUF1.

Based on the functional annotations of the identified proteins, available at Tritryp repository (https://tritrypdb.org), Leish-ESP database (http://leish-esp.cbm.uam.es) and Wikidata platform (https://www.wikidata.org/), Fig. 2 shows a chart informing on the functional categories in which the 90 PUF1-interacting proteins are grouped.

## 2 Experimental Design, Materials and Methods

### 2.1 LiPUF1 antibody purification

A rabbit polyclonal α-LiPUF1 antibody (raised against the *Leishmania infantum* PUF1 recombinant protein [1]) was purified by protein ligand affinity chromatography, following the protocol described by Courey et al. [2], with some modifications noted here. First, as ligand, the MBP-LiPUF1 recombinant protein [1] was dialyzed against PBS 0.1 ×, quantified by Bradford's method [3], lyophilized and suspended in sodium phosphate buffer pH 8.0 at a concentration of 650 μg/ml. Three ml of Affi-Gel 10 beads (Biorad) were washed three times with deionized H_2_O, mixed with the protein solution (2 ml of beads per ml of ligand solution) and incubated overnight on a rocker at room temperature (RT). Beads were separated by filtration and the filtrate was saved and quantified again by Bradford's method. After washing with PBS, the beads were incubated with 100 mM ethanolamine overnight at RT, on a rocker. The resin (affinity matrix with linked MBP-LiPUF1) was washed with PBS and stored in PBS at 4 ᵒC, until use. Afterwards, the resin was incubated with 1.5 ml of the anti-LiPUF1 polyclonal serum for 2 h at RT with shaking. After pouring the mixture into a chromatography disposable column and extensively washing with PBS, the antibodies bound to the resin were eluted with 1M glycine (pH 3).

### 2.2 Leishmania cell culture and protein extraction

Promastigotes of the *L. major* Friedlin strain were grown at 27 °C in M199 medium (Sigma-Aldrich) containing 10% heat-inactivated fetal bovine serum, 100 U/ml penicillin G and 0.1 mg/ml streptomycin sulphate. Mid-log-phase promastigotes (around 1.5 × 10^7^ per ml) were washed twice and suspended in cold PBS to a final concentration of 2.5 × 10^8^ cells/ml. Cell suspension was placed in a 60-mm Petri dish and on an ice tray for UV crosslinking: the cells were UV-irradiated twice with 400 mJ/cm^2^ in a GS Gene Linker chamber (Biorad) at 4 cm distant from the UV source, the pulses were separated by two minutes interval. After UV-irradiation, cells were collected by centrifugation (800 g, 15 min at 4 °C) and lysed by repeated pipetting using 200 µl of a solution containing 10 mM Tris-HCl (pH 7.4), 0.1% (w/v) Nonidet P40 (Roche), 2 mM DTT, 2 mM vanadyl ribonucleoside complexes (Sigma-Aldrich), EDTA-free protease inhibitor cocktail (Roche), 1 mM PMSF, 100U/ml RNaseOUT (Invitrogen) in DEPC-treated water. The lysate was frozen at −80 °C until use. Before using in the immunoprecipitation assays, the lysate was treated with DNase I at 37 °C for 15 min and cleared by centrifugation at 14,000 g for 20 min at RT. This procedure was used for preparing the replicates 1 and 2 (and control-1, see below). For replicate 3 (and control-2), a procedure for enrichment of cytosolic proteins was used. After in vivo crosslinking (above), 2.5 × 10^8^ cells were pelleted and suspended by pipetting in 250 µl of a solution containing 100 mM Tris-HCl (pH 7.4), 20 mM KCl, 12.5 mM MgCl_2_, 0.5% (w/v) Nonidet P40 (Roche), 2 mM DTT, 2 mM vanadyl ribonucleoside complexes (Sigma-Aldrich), EDTA-free protease inhibitor cocktail (Roche), 1 mM PMSF and 100U/ml RNaseOUT (Invitrogen), prepared in DEPC-treated water. The lysate was centrifuged at 800 g for 2 min and 200 µl of the supernatant (cytosolic enriched-fraction) was frozen at −80 °C until use. The discarded pellet was enriched in nuclear proteins as determined by SDS-PAGE and western blot analyses (data not shown).

### 2.3 Immunoprecipitation with the anti-PUF1 antibody

To decrease unspecific binding of proteins to the chromatography beads, *Leishmania* protein extracts (above) were pre-cleaned by incubating with 50 μl of Dynabeads Protein A (Invitrogen), previously incubated with the pre-immune serum. For this purpose, beads were washed twice with 200 μl PBS-0.02% Tween pH 7.4 (PBST) and incubated for 45 min at RT with 150 µl of preimmune serum at 1:50 dilution in PSBT. Unbound antibodies were removed by three washes in 500 µl of PBST, and 200 µl of the *L. major* protein lysate were incubated with the beads for 40 min at 4 °C on a rotor. Non-bound solution (pre-cleaned extracts) was saved for the immunoprecipitation experiments. Then, 100 µl of clean Dynabeads Protein A were washed with 200 µl of PBST and incubated with the affinity-purified anti-PUF1 antibody (1:5 dilution in PBST) for 45 min at RT. As a negative control, parallel incubation of beads with preimmune serum (obtained from the same rabbit, just before PUF1 inoculation) was used. After two washes with PBST, the pre-cleaned lysates were incubated with the antibody-bound beads for 90 min at 4 ⁰C. Afterwards, beads were extensively washed with PBST (five washes) and the proteins were eluted by addition of 2 × Laemmli sample buffer (4% SDS, 20% glycerol, 10% 2-mercaptoethanol, 0.004% bromophenol blue and 0.125 M Tris-HCl, pH 6.8) and heating for 10 min at 90 ᵒC. These samples were analysed by mass-spectrometry.

### 2.4 In-gel digestion of protein samples

The immunocomplexes, eluted in Laemmli buffer (above), were loaded onto a conventional SDS-PAGE gel (10% polyacrylamide). After a brief electrophoresis, when the proteins present in the samples became concentrated in the stacking/resolving gel interface, the gel was staining with Coomassie Brilliant Blue R-25 as described elsewhere [4]. The part of the gel containing the proteins were cut into 1-mm^3^ pieces. Afterwards, the pieces were destained in 50% acetonitrile (ACN). Next, the samples were incubated for 1 h at 56 ᵒC with 10 mM dithiothreitol (DTT) to reduce disulfide bonds and free the thiol groups; then, thiol groups were alkylated with 50 mM iodoacetamide for 1 h at room temperature in darkness. Afterwards, proteins were digested in-gel with 10 ng/μl sequencing grade modified trypsin (Promega, Madison, WI, USA) overnight at 37 °C, as described by Shevchenko et al. [5]. The gel pieces were dehydrated by repeated washing in 100% ACN. Finally, after pipetting out the excess of ACN, shrunk by removing water using sufficient ACN. Acetonitrile was pipetted out and the gel pieces were dried in a Speedvac. The dried gel pieces were reconstituted in 50 mM ammonium bicarbonate pH 8.8; then, 60 ng/µl trypsin was added. The tubes were kept in ice for 2 h and incubated at 37 ᵒC for 12 h. Digestion was stopped by the addition of 1% trifluoroacetic acid solution, followed by a 60 min of incubation on ice. Peptides were purified using OMIX C18 Pipette tips (Agilent Technologies), according to the manufacturer's protocol. Peptides were eluted with 25 μL of 80% ACN containing 5% formic acid, and the solvent was removed using a Speedvac evaporator. Finally, peptides were reconstituted in 10 μL of 0.1% formic acid prior to LC−MS/MS analysis.

### 2.5 Reverse phase-liquid chromatography RP-LC-MS/MS analysis

Ten μl of the desalted proteins (in 0.1% formic acid solution, above) were separated by RP-LC-MS/MS in an Easy-nLC II system coupled to an ion trap LTQ-Orbitrap-Velos-Pro hybrid mass spectrometer, equipped with a nanospray flex ion source (Thermo Scientific). The peptides were concentrated (on-line) by reverse phase chromatography using a 0.1mm × 20 mm C18 RP precolumn (Thermo Scientific), and then separated using a 0.075mm × 25 cm C18 RP column (Thermo Scientific) operating at 300 nl/min. Peptides were eluted using a 180-min dual gradient from 5 to 25% solvent B in 135 min followed by gradient from 25 to 40% solvent B over 180 min (Solvent A: 0.1% formic acid in water, solvent B: 0.1% formic acid, 80% acetonitrile in water). Electrospray ionization (ESI) was performed using a Nano-bore emitters Stainless Steel ID 30 μm (Proxeon) interface. The Orbitrap resolution was set at 30.000. Peptides were detected in survey scans from 400 to 1600 amu (1 μscan), followed by twenty data dependent MS/MS scans (Top 20), using an isolation width of 2 u (in mass-to-charge ratio units), normalized collision energy of 35%, and dynamic exclusion applied during 30 s periods.

Peptide identification from raw data was carried out using PEAKS Studio X**+** search engine (Bioinformatics Solutions Inc., Waterloo, Ontario, Canada). Database search was performed against Uniprot *L. major* predicted proteins (30/09/19; 8038 entries). The following constraints were used for the searches: tryptic cleavage after Arg and Lys, up to two missed cleavage sites. The precursor tolerance was 20 ppm and 0.6 Da for MS/MS fragment ions. Modifications by carbamidomethylation of cysteine and oxidation of methionine were considered as variable. False discovery rates (FDR) for peptide spectrum matches (PSM) were limited to 0.01. Peptides having a minimum sequence length of 6 amino acid residues were considered. Only those proteins identified by at least two distinct peptides and at least one unique peptide were considered as reliably identified.

The mass spectrometry proteomics data have been deposited to the ProteomeXchange Consortium via the PRIDE [6] partner repository with the dataset identifier PXD022581 and 10.6019/PXD022581.

### 2.6 Data and statistical analysis

Proteins from the different independent co-immunoprecipitation experiments (three replicates with anti-PUF1 antibody and two control replicates) were analysed taking into account the number of peptides found for every protein. Also, proteins were considered only if they were detected in at least two out to the three immunoprecipitation replicates performed with the anti-PUF1 antibody. Afterwards, for each protein, the number of peptides identified, considering the three anti-PUF1 immunoprecipitation assays, was summed up, and the same for the peptides identified in the control samples. The statistical significance was analysed by a binomial distribution test; only differentially distributed proteins, supported by p-values lower than 0.05, were considered as putative PUF1-interactors (see Supplementary Table S3). A functional classification (Fig. 2) of the selected proteins was performed based on the information available in specific databases: TriTrypDB (https://tritrypdb.org/tritrypdb/), Leish-ESP (http://leish-esp.cbm.uam.es) and Wikidata platform (https://www.wikidata.org/). Additionally, updated information published in recent articles [7], [8], [9] was incorporated to the protein entries identified in this analysis.

## CRediT Author Statement

**África Sanchiz:** Data curation, Writing- Original draft preparation. **Darío López-García:** Data curation. **Carlos García-García:** Proteomics analyses, Software, Writing- Reviewing. **Irene Ozaez:** Immunoprecipitation assays. **Begoña Aguado:** Funding, Reviewing, Editing. **Jose María Requena:** Conceptualization, Funding, Writing- Reviewing and Editing.

## Declaration of Competing Interest

The authors declare no conflict of interest. The authors declare that they have no known competing financial interests or personal relationships which have, or could be perceived to have, influenced the work reported in this article.

## Appendix Supplementary materials

Image, application 1

Image, application 2

Image, application 3
